# Molecular characterization of subcellular localization and nucleocytoplasmic shuttling of PRV UL54

**Published:** 2011-01-10

**Authors:** Meili Li, Shuai Wang, Junji Xing, Hong Guo, Chunfu Zheng

**Affiliations:** 1 Wuhan Institute of Virology, Chinese Academy of Sciences, Wuchang, Wuhan 430071, PR China

## 

Pseudorabies virus (PRV) UL54 protein localized almost exclusively to the nucleolus. By constructing a series of mutants, the putative nuclear localization signal (NLS), nucleolar localization signal (NoLS) and nuclear export signal (NES) of UL54 were for the first time mapped to amino acids ^45^RRRRGGRGGRAAR^57^, ^61^RQRRR^65^ and ^240^LQNLRLKLGPFL^251^, respectively. In addition, nuclear localization of UL54 was important for its transcriptional regulation of glycoprotein C promoter. The nuclear import of UL54 was abrogated by dominant negative RanGTP and importin β1, respectively, indicating that UL54 targeted to the nucleus by means of a classic Ran- and importin β-dependent nuclear import mechanism. Heterokaryon assays demonstrated that UL54 was a nucleocytoplasmic shuttling protein and this property could not be blocked by leptomycin B, an inhibitor of the chromosome region maintenance 1 (CRM1). However, ectopic expression of the mRNA export receptor TAP(NXF1) promoted the nuclear export of UL54 and interacted with UL54, suggesting that UL54 shuttles between the nucleus and the cytoplasm via a TAP(NXF1), but not CRM1, dependent nuclear export pathway. The present study demonstrated that UL54 is a nucleolar protein, adding UL54 to the growing list of transactivators which localize to the nucleolus and shuttle between the nucleus and the cytoplasm.

